# Metal Organic Framework Derived MnO_2_-Carbon Nanotubes for Efficient Oxygen Reduction Reaction and Arsenic Removal from Contaminated Water

**DOI:** 10.3390/nano10091895

**Published:** 2020-09-22

**Authors:** Vadahanambi Sridhar, Inwon Lee, Kwang Hyo Jung, Hyun Park

**Affiliations:** 1 Global Core Research Centre for Ships and Offshore Plants (GCRC-SOP), Pusan National University, Busan 46241, Korea; sridhar@pusan.ac.kr; 2 Department of Naval Architecture and Ocean Engineering, Pusan National University, Busan 46241, Korea; inwon@pusan.ac.kr (I.L.); kjung@pusan.ac.kr (K.H.J.)

**Keywords:** metal organic frameworks, manganese oxide, Oxygen Reduction Reaction (ORR), microwave synthesis, carbon nanotubes, arsenic removal

## Abstract

Even though manganese oxides are attractive materials for batteries, super-capacitors and electro-catalysts for oxygen reduction reactions, in most practical applications MnO_2_ needs to be hybridized with conductive carbon nano-structures to overcome its inherent poor electrical conductivity. In this manuscript we report microwave-assisted synthesis of MnO_2_ embedded carbon nanotubes (MnO_2_@CNT) from Mn-H_3_BTC (benzene-1,3,5-carboxylic acid) metal organic frameworks (MOF) precursors. Using graphene oxide as microwave susceptible surface, MnO_2_ nano-particles embedded in three dimensional reduced graphene oxide (rGO) -CNT frameworks (MnO_2_@CNT-rGO) were synthesized which when applied as electro-catalysts in oxygen reduction reaction (ORR) demonstrated comparable half-wave potential to commercial Pt/C, better stability, and excellent immunity to methanol crossover effect in alkaline media. When carbon fiber (CF) was used as substrate, three-dimensional MnO_2_@CNT-CF were obtained whose utility as effective adsorbents for arsenic removal from contaminated waters is demonstrated.

## 1. Introduction

One of the major obstacles in the commercial development of fuel cells is the sluggish oxygen reduction reaction (ORR) at the cathode. Platinum group metals (PGM) such as ruthenium, palladium, iridium and especially platinum (Pt) are the most current widely used active ORR catalysts, but have serious disadvantages like low methanol tolerance, scarcity (by weight Pt constitutes only 0.003 ppm in the Earth’s crust and mere 172 tonnes of Pt were produced in 2016) and its high cost (24,000 United States Dollars (USD) per kg) prevents it to be a sustainable, economical and long-term electro-catalyst for ORR reaction [1]. Therefore, there have been intensive efforts by researchers to develop low-cost and durable non-PGM electro-catalysts. These efforts resulted in promising ORR catalysts which can be broadly classified into three categories: non-PGM metal catalysts [2], metal-free catalysts especially hetero-atom doped carbons [3] and hybrids of non-PGM catalysts with carbon nano-structures [4]. Amongst these three, the third type of carbon-metal hybrids which consists of nano sized non-PGM metal compounds of iron [5], cobalt [6], manganese [7] etc. embedded/dispersed/anchored on nano scaled carbons such as graphene [8], carbon nanotubes [9] and carbon nanofibers [10] have rapidly evolved as attractive alternatives to PGM based ORR electro-catalysts. Among these, manganese is worth special mention since manganese is the fifth most abundant metal and constitutes 0.1 wt% of the Earth’s crust [11] and manganese compounds like oxides [12,13], nitrides [14], sulfides [15], phosphates [16] etc. have rapidly emerged as suitable materials for energy storage applications like lithium [17], sodium-ion batteries, super-capacitors, dye sensitized solar cell (DSSC) [18], catalyst in ORR reaction [19] etc. Manganese oxides (Mn_x_O_y_), especially manganese dioxide MnO_2_, exhibiting various morphologies like nano-particles [20], nanotubes [21], nano-belts [22], nano-fibers [23], nano-rods [24], nano-wires [25], nano-flowers [26], nano-urchins [27], nano-plates [28], nano-disks [29], etc. synthesized by either sol-gel [30], reduction [31], co-precipitation [32], hydrothermal [33] etc. has proven as an excellent material in a wide range of energy storage [34], energy generation [35] and water treatment applications [36]. Due to its low electrical conductivity, MnO_2_ itself is seldom used in ORR reactions and has to be hybridized with conductive materials especially conductive carbon black [37], graphene [38], carbon nanotubes (CNTs) [39], etc. which is usually done either by in situ or ex situ techniques. In ex situ techniques, pre-synthesized MnO_2_ nano-particles are mixed with functionalized carbon substrates whereas in in situ techniques, MnO_2_ nano-particles are synthesized either by sol-gel [40], hydrothermal [41], electrochemical reduction [42] of manganese salts in solutions in which nano-carbons like graphene or CNTs are dispersed. Despite being widely used, the above-mentioned techniques have disadvantages like agglomeration of MnO_2_ nano-particles due to Ostawald ripening and necessity of covalent functionalization of carbon substrate which reduces its electrical conductivity. In order to overcome these practical difficulties there is a need for one-step synthesis of MnO_2_ nano-particles dispersed, anchored or hybridized with conductive carbon substrates. Metal organic frameworks (MOF), zeolitic imidazolate frameworks (ZIF) and covalent organic frameworks (COF) are rapidly emerging as attractive precursors for synthesis of meso-porous nanostructures. A wide range of nanostructures such as zero-dimensional quantum dots [43], one dimensional carbon nanotubes [44,45], metal nanorods [46], metal nano-wires [47], carbon nano-fibers [48] and two-dimensional nano-sheets [49] etc. have been synthesized. Herein, we report synthesis of MnO_2_ nano-particles embedded in carbon nanotubes using Mn-H_3_BTC (benzene-1,3,5-carboxylic acid) metal organic framework (MOF) as the single source of both CNT and MnO_2_. Using graphene oxide as microwave susceptible surface, MnO_2_ nano-particles embedded in carbon nanotubes-reduced graphene oxide three dimensional, mesoporous frameworks (MnO_2_@CNT-rGO) were obtained which were applied as electro-catalysts for ORR reaction. More ever by using carbon fibers as the microwave absorbers, MnO_2_ nano-particles embedded in carbon nanotubes anchored on carbon fibers (MnO_2_@CNT-CF) were obtained and its utility in removal of arsenic from contaminated water is reported.

## 2. Experimental

### 2.1. Materials and Methods

Graphite (99.5% pure, ES 350 F5) was procured from Samjung C&G, Gyeongsan, Korea whereas reagent grade sulfuric acid (CAS Number: 7664-93-9; H_2_SO_4_), hydrochloric acid (CAS Number: 7647-01-0; HCl), sodium nitrate (CAS Number: 7631-99-4; NaNO_3_), hydrogen peroxide (CAS Number: 7722-84-1; H_2_O_2_), potassium permanganate (CAS Number: 7722-64-7; KMnO_4_), benzene-1,3,5-carboxylic acid (CAS Number: 554-95-0; H_3_BTC) and manganese acetate (CAS Number: 638-38-0; (CH_3_CO_2_)_2_Mn) were purchased from Sigma-Aldrich, Seoul, Korea and were used as received. Microwave irradiation was carried out on Daewoo Korea KR-B202WL microwave oven (Daewoo, Seoul, Korea) operating at 700 W power and frequency of 2450 MHz. Morphologies of the ‘uncoated’ samples was tested using a Nova Nano SEM 230 FEI Field-emission scanning electron microscopy (FE-SEM, Seoul, Korea) in both in-lens and secondary electron imaging mode. TALOS F200X high-resolution transmission electron microscopic (HRTEM) (Thermo Fisher Scientific Korea Ltd., Seoul, Korea) operating at 200 kV was used to record high and low resolution images; high-angle annular dark-field (HAADF) images and elemental Energy Dispersive Spectroscopy (EDS) maps. High-resolution XPS (X-ray photoelectron spectroscopy) was used to perform chemical analysis on a Sigma Probe Thermo VG spectrometer (Thermo Fisher Scientific Korea Ltd., Seoul, Korea) using Mg K_α_ X-ray sources. The freeware XPSPEAK version 4.1 was used to curve fit XPS data. BET surface area was measured by nitrogen adsorption and desorption isotherms at 77 K using a BEL Japan Inc. Belsorp Mini II Surface Area (Microtrac MRB, York, PA, USA).

### 2.2. Electrochemical Measurements

All the electrochemical measurements were conducted on a CHI 660 electrochemical station (CH Instruments, Inc., Austin, TX, USA) with a conventional three-electrode system as reported in our previous work [43]. In a typical electrochemical measurement, a total of 5 mg of MnO_2_@CNT-rGO was sonicated for 30 min in 500 mL solution consisting of 480 mL isopropyl alcohol and 20 mL of 5 wt% nafion solution for 30 min. A total of 12 mL of the above well dispersed suspension was dropped onto a 5 mm diameter glassy carbon rotating disk electrode (RDE) or rotating ring-disk electrode (RRDE, 4.93 mm inner diameter and 5.38 mm outer diameter) for two times and air dried naturally, fixed with a total loading mass of about 0.6 mg/cm^2^. 0.1 M KOH aqueous solution was used as the electrolyte whereas counter electrode was saturated calomel electrode (SCE) and Pt wire served as the reference electrode. Prior to use, the electrolyte was saturated with oxygen which was continuously supplied during the experimental operation. All potential values reported in this study were converted to the reversible hydrogen electrode (RHE) scale, according to the equation: E_RHE_ = ES_CE_ + 0.0591pH + 0.242. For the stability test, the working electrode ran at –0.7 V vs. SCE for 50,000 s in O_2_-saturated 0.1M KOH with a rotation rate of 1200 rpm. For comparison, commercial Pt/C (Sigma-Aldrich, Seoul, Korea) (20 wt% Pt) powder purchased from was tested under the same conditions, with a loading mass of 0.5 mg cm^−2^. Linear sweep voltammetry (LSV) was measured by RDE/RRDE technique (CH Instruments, Inc. Austin, TX, USA) the with the scan rate of 10 mV/s at various rotating speeds from 400 to 2400 rpm.

### 2.3. Arsenic Removal Tests

In order to study the rate of arsenic removal by novel MnO_2_@CNT-CF adsorbents, batch adsorption tests of As(III) aquous solutions prepared from sodium arsenite (NaAsO_2_) were carried out. Experiments were carried out in 250 mL flasks with 5 mg of MnO_2_@CNT-CF adsorbents and 100 mL of arsenic solution at room temperature (25 °C).

### 2.4. Synthesis of MnO_2_@CNT-rGO

**Figure 1:**
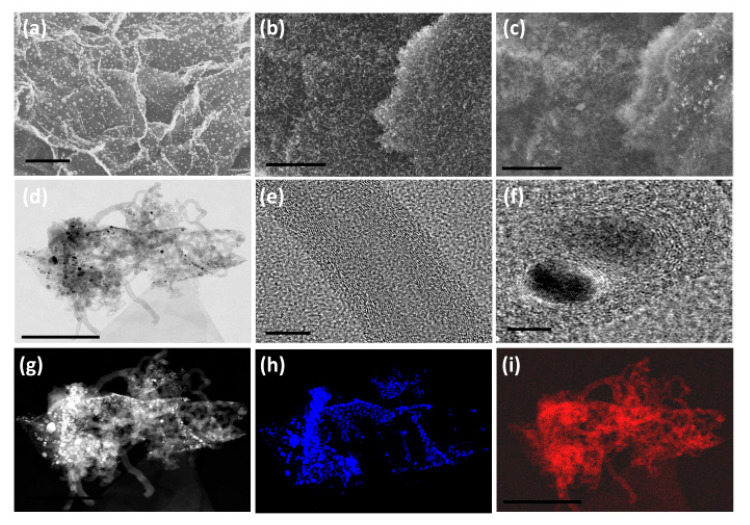
In-lens SEM micrographs (**a**,**b**) of Mn-H_3_BTC decorated graphene oxide and MnO_2_@CNT-rGO synthesized from the same by microwave synthesis and its corresponding secondary electron image (**c**). High-resolution transmission electron microscopic (HRTEM) image of MnO_2_@CNT-rGO (**d**) and high magnification image of defect rich individual carbon nanotubes (**e**) and MnO_2_ nanoparticle (**f**); high-angle annular dark-field (HAADF) image (**g**) and its corresponding manganese (**h**) and carbon map (**i**). Scale bars are 1 µm in (**a**–**c**); 500 nm in (**d**); 50 nm (**e**); 20 nm (**f**); and 500 nm in (**g**–**i**).

Manganese based metal-organic framework (Mn-MOF) were prepared by coordination reaction between manganese acetate and 1,3,5-benzenetricarboxylate wherein 0.4 g of manganese acetate (2.5 mmol) was dispersed in ethyl alcohol solution to form solution A, whereas 0.525 g of 1,3,5-benzenetricarboxylate (BTC, equal to 2.5 mmol) was dissolved in ethyl alcohol to form solution B. Subsequently these two solutions A and B were mixed with ethanolic graphene oxide dispersion (0.5 g/L) made by a modified Tour’s method [50] and mixed in an ultrasonic bath for 60 min. Subsequently this solution was dried in an oven to yield Mn-H_3_BTC (benzene-1,3,5-carboxylic acid) metal organic frameworks (MOF) decorated graphene oxide (representative SEM micrograph shown in Figure 1a and representative XRD in Figure S1 of Supplementary File) which was subsequently transferred to a glass vial and subjected to microwave radiation in a microwave oven, model number: KR-B202WL with output power of 700 W and input power of 1120 W operating at 2450 MHz at 700 W for 300 s to form a fluffy powdery solid. The obtained product was washed alternately with ethanol and De-ionized (DI) water to remove any unreacted BTC and finally dried in an oven at 100 °C for 60 min to yield MnO_2_ nano-particles embedded in carbon nanotubes anchored on reduced graphene oxide three dimensional frameworks (MnO_2_@CNT-rGO).

## 3. Results and Discussion

The morphology and microstructure of the synthesized MnO_2_@CNT-rGO as studied by in-lens (Figure 1b) SEM micrograph exhibits extensive and very high density growth of micrometer long carbon nanotubes on reduced graphene oxide surface whereas the corresponding secondary electron image (Figure 1c) shows the relatively brighter MnO_2_ nano-particles well dispersed amongst the dense carbon nanotubes which appear less brighter when compared to the reduced graphene oxide substrate. Representative low resolution TEM micrograph (Figure 1d) confirms the presence of micrometer long, multi-walled carbon nanotubes anchored on the walls and edges of reduced graphene oxide substrate and MnO_2_ nano-particles are dispersed predominantly amongst the CNT either enclosed inside or anchored along the walls of CNT. Careful observation by HRTEM (Figure 1e) reveals that nanotubes are defect rich with the outer walls more prone to defects whereas the inner walls are well defined, relatively defect free and highly crystalline. The CNTs synthesized from our novel precursor, Mn-H_3_BTC, have 18–26 layers with the inner walls showing a d-spacing of 0.34 nm nearly identical with that of (002) planes of graphite whereas the d-spacing amongst the outer 6-8 walls is slightly higher at 0.36 nm spacing. The manganese oxide nano-particles are highly crystalline (Figure 1f) and have lattice fringe spacing of ~0.28 nm which is corresponds to (111) of λ-MnO_2_ [51].

Some manganese nano-particles also exhibit core-shell structure consisting of MnO_2_ nano-particle cores encapsulated in well-defined but defect rich multi-layered graphene shells. The mechanism of formation of carbon nanotubes and core-shell MnO_2_ nano-particle can be explained in two concurrent steps. In the first step, under microwave radiation, Mn-H_3_BTC MOFs anchored on graphene oxide start to thermally decompose to manganese nano-particles which get anchored on the point and dislocation defects generated on chemically synthesized graphene oxide. With further increase in microwave radiation, the benzene 1,3,5-carboxylic acid, H_3_BTC, thermally degrades to hydrocarbons by free radical mechanism by the following three steps: in the first step of degradation, H_3_BTC, one molecule of COOH is eliminated to form benzoic acid. This benzoic acid is either turned into phenol by elimination of CO or benzaldehyde by elimination of OH. These phenols and aldehydes are can thermally decompose to simpler mono-aromatic ringed compounds like toluene or styrene or complex multi-ringed compounds like 1,1-biphenyl or benzophenone by polymerization of some radicals [52]. This decomposition and formation of hydrocarbons is further catalyzed by the MnO_2_ nano-particles anchored on the graphene substrates [53]. With further increase in microwave radiation, these hydrocarbon moieties are vaporized and are captured by the manganese nano-particles which catalyze dehydrogenation reactions thus leading to the formation of multi-walled carbon nanotubes.

**Figure 2:**
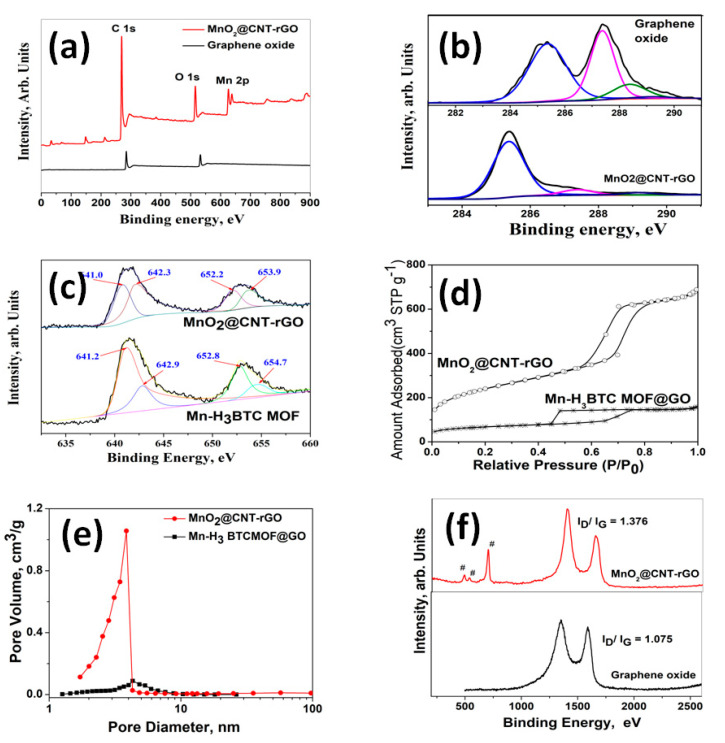
XPS survey spectra of MnO_2_@CNT-rGO and graphene oxide (**a**); deconvoluted C1 s spectra of MnO_2_@CNT-rGO and graphene oxide (**b**); deconvoluted Mn 2s spectra of MnO_2_@CNT-rGO and Mn-H_3_BTC MOF (**c**); nitrogen adsorption isotherms (**d**) and pore size distribution (**e**) of MnO_2_@CNT-rGO and Mn-H_3_BTC MOF; and Raman spectra of MnO_2_@CNT-rGO and GO (**f**).

The variations in compositional and electronic states of graphene oxide (GO) and MnO_2_@CNT-rGO, detailed X-ray photoelectron spectroscopy (XPS) studies were carried out and the survey scans are plotted in Figure 2a. In case of GO, the survey scan shows two sharp peaks centered at ~284 and ~586 eV corresponding to C1s and O1s moieties, whereas the plot of MnO_2_@CNT-rGO consists of an additional peak centered at 642 to 651 eV corresponding to Mn 2p^3/2^ and Mn 2p^1/2^ of manganese moieties. Another interesting observation is the substantial increase in carbon to oxygen ratio from 0.989 in GO to 1.587 in MnO_2_@CNT-rGO which gives credence to our contention that under microwave radiation, not only CNTs are grown from H_3_BTC, but also the graphene oxide substrate is reduced to reduced graphene oxide (rGO). More proof of this can be observed from deconvoluted C1s XPS spectra of GO and MnO_2_@CNT-rGO plotted in Figure 2b. The C1s of GO plotted in bottom half of figure shows peaks centered at ~285.1 and 287.14 eV corresponding to carbonyl (C-O) and carboxyl (C=O) moieties of graphene oxide whereas the minor peak at 288.8 eV is attributed to carboxylic (O=C-O) groups [54]. However, in MnO_2_@CNT-rGO a broad peak centered at 284.5 eV corresponding to sp^2^ carbon (C=C) is dominating with minor peaks at 286.4, 288.8 and 291.5 eV attributed to residual epoxides [55], carboxylic and plasmon resonance of valence electrons of rGO [56]. In order to study the ionic states of manganese moieties in Mn-H_3_BTC MOF and MnO_2_@CNT-rGO, high-resolution XPS spectroscopy was carried out and the deconvoluted Mn 2p of the two samples is plotted n Figure 2c. In case of Mn-H_3_BTC MOF, two distinct peaks at 641.96 and 650.8 eV corresponding to Mn 2p^3/2^ and Mn 2p^1/2^ with a peak distance of 8.84 eV is observed whereas in MnO_2_@CNT-rGO the peaks are centered at 641.66 and 653.4 eV with a substantially higher peak distance of 11.74 eV. Though a suitable explanation for this substantially higher spin orbit splitting ΔE, is elusive but it can be attributed to tunable spin–orbit coupling [57] of MnO_2_ moieties anchored on three-dimensional graphene–CNT frameworks. The microwave transformation of Mn-H_3_BTC MOF anchored on graphene oxide to MnO_2_@CNT-rGO not only results in substantial chemical changes as evident from XPS studies but also results in enhanced physical properties especially increased surface area as reflected in the N_2_-adsorption isotherm plotted in Figure 2d. In case of Mn-H_3_BTC MOF anchored on graphene oxide, the surface area very low and is in the range of 80 m^2^ g^−1^ whereas the nitrogen adsorption isotherm of MnO_2_@CNT-rGO shows type I/II characteristic with surface area in the range of 596 m^2^ g^−1^. This fivefold increase in surface area is due to “spacer” functionality of vertically anchored carbon nanotubes, which hinders the inter-laminar van der Waals forces of attraction between adjacent graphenic layers. The pore size distributions of Mn-H_3_BTC MOF anchored on graphene oxide and of MnO_2_@CNT-rGO are plotted in Figure 2e. In case of Mn-H_3_BTC MOF anchored on graphene oxide, the maximum pore diameters are centered from 4 to 7 nm whereas in MnO_2_@CNT-rGO the maximum pore size distribution is predominantly below 4 nm which indicates that the growth of carbon nanotubes on graphene substrate increases the meso-porosity [58].

Raman spectroscopy was used to study the structural changes of graphene oxide and MnO_2_@CNT-rGO (Figure 2f). In the Raman spectra of graphene oxide plotted only two peaks the D band (attributed to the disorder induced phonon mode vibrations of graphene) and the G band (associated with the first-order E_2_g mode scattering of sp^2^ carbon atoms in graphitic domain), respectively, appear at 1349 and 1590 cm^−1^, respectively. In our H_3_BTC MOF derived MnO_2_@CNT-rGO, besides the two carbon related peaks, an intense peak at 651 cm^−1^ attributed to manganese oxides [59] with minor but visually discernible peaks at 471 and 516 cm^−1^ corresponding to A_1_g and Eg vibration modes of manganese oxide moieties [60] can also be observed. More ever, there is a substantial increase in the I_D_/I_G_ ratio (ratio of intensities of D band to G band) from 1.075 in graphene oxide to 1.376 in MnO_2_@CNT-rGO which suggests partial healing of the in-plane defects generated on graphene oxide during oxidation by the growth of carbon nanotubes.

**Figure 3:**
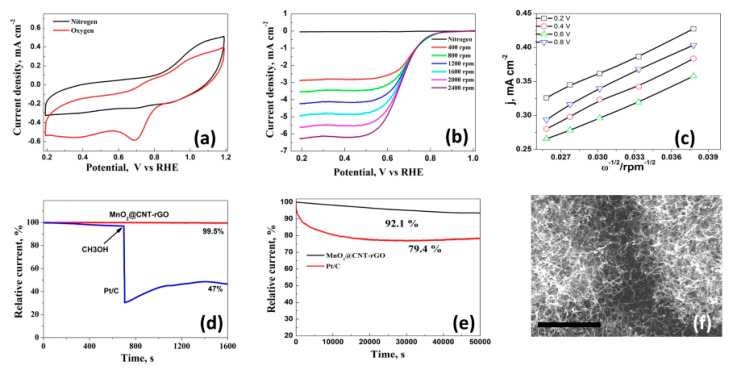
Cyclic voltammograms (CV) of MnO_2_@CNT-rGO electrodes in 0.1 M KOH saturated with N_2_ (black) and O_2_ (red) at a sweep rate of 50 mVs^−1^ (**a**); linear sweep voltammetry (LSV) curves of MnO_2_@CNT-rGO in 0.1 M KOH at rotation speeds ranging from 400 to 2400 rpm (**b**); corresponding K–L plots of MnO_2_@CNT-rGO at various potentials (**c**); Methanol tolerance test of MnO_2_@CNT-rGO and commercial Pt/C (**d**); variation in residual current with time in MnO_2_@CNT-rGO and commercial Pt/C (**e**) and post-mortem SEM micrograph of (**f**) of MnO_2_@CNT-rGO after cycling in 0.1 M KOH (**f**), scale bar is 1 µm.

The cyclic voltammetry behavior of MnO2@CNT-rGO was conducted between 1.2 and 0.2 V vs. RHE in nitrogen and oxygen saturated 0.1 M KOH electrolytes and the plot is shown in Figure 3a. In nitrogen saturated electrolyte the CV curve was devoid of any redox peaks whereas in oxygen saturated solutions, a cathodic peak (E_peak_) at 0.72 V and its onset (E_onset_) at 0.89 V associated with electro-catalytic reduction of oxygen by the manganese moeities of MnO_2_@CNT-rGO is evident which is in line with other reported values in manganese based ORR electrodes as shown in the following Table 1 below:

To further study the electrocatalytic kinetics of MnO_2_@CNT-rGO in ORR, polarization curves measured with different rotation speed were tested. With the increase of rotation speed, diffusion limiting current density increases due to the shorter O_2_ diffusion length as evident from Figure 3b. The Koutecky–Levich plots recorded at various potentials in oxygen saturated 0.1 M KOH electrolyte is shown in Figure 3c after subtracting the current background in N_2_-saturated solution. At all the investigated potential voltages, plots exhibit excellent linearity and almost similar slopes, indicating that first-order reaction kinetics is followed in ORR and the applied potential has no influence on the number of electrons transferred (n) which is almost constant at 3.90 to 3.92. This value close to the ideal value of 4 indicates that the electro-catalytic reaction proceeds in a direct pathway involving direct reduction of oxygen to water. Another important factor that governs the efficiency of ORR electro-catalyst is its methanol tolerance because in most practical fuel cells, the methanol fuel will permeate from anode to cathode through the polymer membrane which causes a drastic reduction in the electro-catalytic performance of the catalyst due to carbon monoxide poisoning. The methanol tolerance of our novel MnO_2_@CNT-rGO and commercial Pt/C was evaluated in 0.1 mol dm^−3^ oxygen saturated KOH solution and data plotted in Figure 3d which shows that in case of Pt/C there was more than half (~47.5%) decrease in current within 700 s whereas in MnO_2_@CNT-rGO this decrease in current is minuscule (less than 0.6%) even after 1600 s. Chrono-amperometric test which is an indicator of durability of an electro-catalyst was performed and compared with commercial Pt/C electrode (Figure 3e) which shows that about 92.1% of initial current was retained even after 50,000 s in MnO_2_@CNT-rGO whereas in Pt/C this retention rate was 79.4%. These excellent ORR properties of MnO_2_@CNT-rGO hybrid in our work are believed to result from the synergistic effect of 3D carbon nanotubes-reduced graphene oxide and the deposited MnO_2_, as well as their intrinsic advantages mentioned above, which could provide more active surface area and accelerate the O_2_ diffusion and electron transfer.

**Figure 4:**
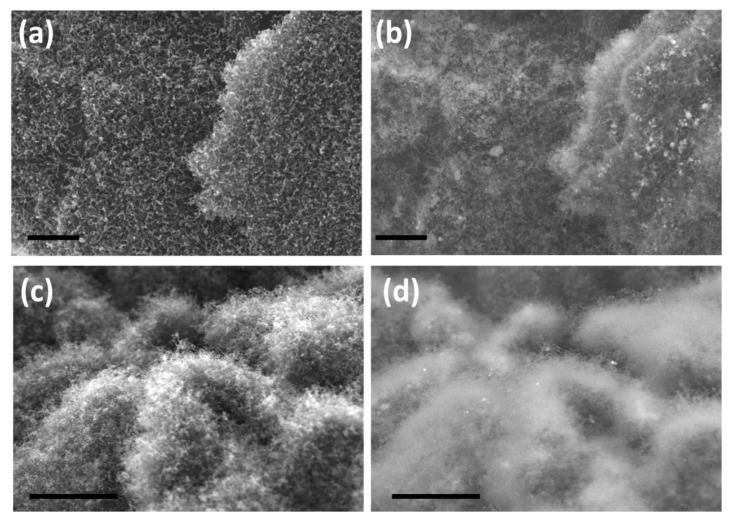
Representative in-lens and their corresponding secondary electron SEM micrographs of H_3_BTC derived carbon nanotubes using cobalt (**a**,**b**) and iron catalysts (**c**,**d**). Scale bar is 1 µm in (**a**,**b**) and 3 µm (**c**,**d**).

In order to test the utility of our newly developed H_3_BTC precursor to grow CNTs by other catalysts, cobalt and iron were chosen and representative SEM micrographs of the same are exhibited in Figure 4. In case of cobalt catalyst, carbon nanotubes of shorter length and less density are grown on reduced graphene oxide substrate (Figure 4a,b) whereas in case of iron based H_3_BTC MOF as the CNT precursors, both density and length of carbon nanotubes is substantially higher (Figure 4c,d). This observation can be attributed to the higher rate of dissolution of carbon in iron when compared to cobalt which leads to good growth of CNT.

**Figure 5:**
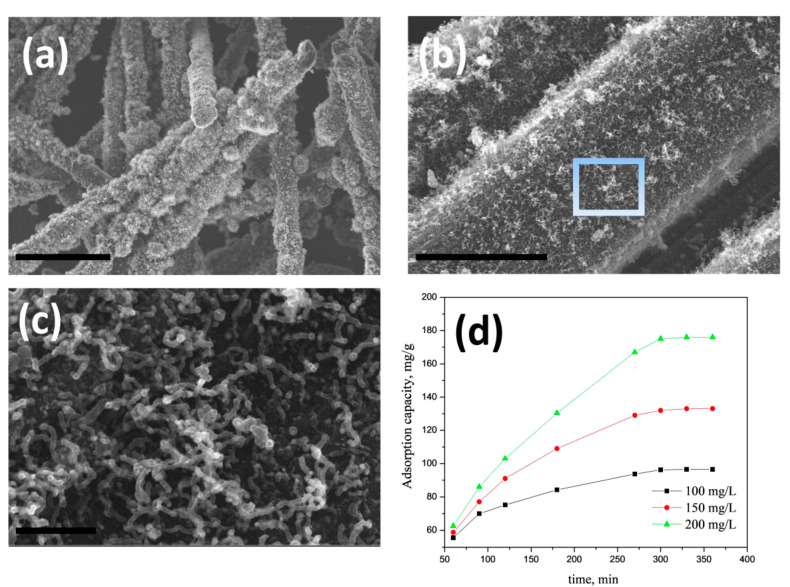
Representative SEM micrographs of MnO_2_@CNT-CF hybrid synthesized by using novel Mn-H_3_BTC precursors at low (**a**) and high (**b**) magnifications; (**c**) is the high-resolution image of the portion highlighted as rectangle in (**b**); scale bars are 30 µm (**a**); 3 µm (**b**) and 500 nm (**c**); Adsorption isotherms of arsenic on MnO_2_@CNT-CF nanostructures at various concentrations (**d**).

We also investigated the utility of Mn-H_3_BTC precursor to grow CNT on other carbonaceous substrates like carbon fiber. The choice of carbon fiber was governed by the fact it is also a microwave susceptible surface and can withstand short-term high-density microwave radiation without undergoing significant structural damage. A similar synthesis procedure as described in Section 2.2 with substitution of graphene oxide dispersion with carbon fiber is used. However, before use the commercially available CFs were washed successively with 10% HCl and DI water to remove any commercial ‘sizing’ materials and dipped in Mn-H_3_BTC stock solution for 30 min which were subsequently removed and dried in oven at 70 °C to obtain Mn-H_3_BTC MOF decorated carbon fibers (representative XRD and SEM in Figures S1 and S2 of Supplementary File, respectively) which were subjected to microwave irradiation for 60 s to obtain high density, MnO_2_ catalyzed CNT grown on carbon fibers (MnO_2_@CNT-CF). The representative SEM micrographs of the same is exhibited in Figure 5a,b indicates that though the growth of CNTs on carbon fibers is clearly visible, but the density of CNT growth is comparatively low due to the fact that intrinsic curvature of fiber surface is not conducive for anchoring of catalyst particles. High-resolution SEM images in Figure 5c shows that the micrometer long carbon nanotubes are formed but distorted when compared to that of graphene anchoring. This result shows the versatility of our newly discovered H_3_BTC based MOF precursors as excellent source to grow CNTs on any microwave susceptible substrates. The applicability of MnO_2_@CNT-CF for the removal of arsenic from contaminated water is investigated. Lead and Arsenic are synonymous with heavy metal pollutants of drinking water and a wide range of adsorbents are investigated for removal of arsenic, amongst which iron-based adsorbents are the most popular and are widely investigated. Manganese can also show equal if not better adsorption capacity for arsenic removal. In this manuscript we report for the first time the utility of manganese oxide-CNT on carbon fibers as possible adsorbent of arsenic. The rate of adsorption at various concentrations by our novel MnO_2_@CNT-CF adsorbent is plotted in Figure 5d which shows that at all investigated pH values, our MnO_2_@CNT-CF nanostructures showed comparable adsorption capacity and when compared with our previously reported Fe@CNT-CF [70]. The well dispersed MnO_2_ nano-particles in open pore hybrid network of carbon nanotubes vertically anchored on carbon fiber substrate increases the active surface area and facilitates for the optimal capture and retention of arsenic moieties. This result proves that a wide range of heavy metal adsorbents can be fabricated using our H_3_BTC based MOF precursor derived carbon nanotubes.

## 4. Conclusions

In summary, we have described microwave synthesis of manganese oxide embedded in carbon nanotubes (MnO_2_@CNT-rGO) obtained from manganese based metal organic frameworks constructed from benzene 1,3,5-carboxylic acid linkers. Structural analysis by SEM and HRTEM reveals that MnO_2_@CNT-rGO possesses a well-defined millimeter long, carbon nanotubes with nanometric MnO_2_ nanoparticles embedded inside the protective shells of tube walls. Due to the synergistic effect of the structural merits and composition, the resulting MnO_2_@CNT-rGO catalyst exhibits excellent ORR catalytic activity exceeding that of commercial Pt/C catalyst with excellent stability and great durability. The excellent ORR catalytic efficiency of our newly developed electro-catalyst combined with its ease of synthesis from relatively low priced and widely available raw materials opens up new opportunities for their application in fuel cells and metal–air batteries. The utility of MnO_2_@CNT-CF for effective removal of arsenic from contaminated water at different pH values is also demonstrated.

## Figures and Tables

**Table 1:** Comparison of oxygen reduction reaction (ORR) performance our electrode with other MnO_2_ anchored/hybridized/embedded in other carbon based materials.

Serial Number	Type of Mn Based Catalyst	Electrolyte	E_onset_ (V)	E_peak_ (V)	Electron Transfer Number, n	Reference
1	MnO_2_ nano-particles anchored on sulphur doped graphene	0.1 M KOH	0.91	0.79	3.95	[61]
2	MnO_2_ nanosheets on nitrogen doped carbon	0.1 M KOH	0.918	0.78	3.9	[62]
3	MnO_2_ nano-particles anchored on reduced graphene oxide	0.1 M KOH	0.847	0.76	3.85	[63]
4	MnO_2_ nano-rods anchored on carbon nitride	0.1 M KOH	0.8	0.74	3.8	[64]
5	MnO nano-particles on nitrogen doped graphene	0.1 M KOH	0.83	0.72	3.03	[65]
6	MnO_2_ nano-films anchored on hollow graphene spheres	0.1 M KOH	0.94	0.73	3.85	[66]
7	Mn_x_Co_y_O_4_ nano-particles anchored on carbon nanotubes	1 M KOH	0.81	0.89	Not reported	[67]
8	MnO_2_ nano-rods anchored on sugar derived carbon nanosheets	0.1 M KOH	0.914	Not reported	3.12	[68]
9	MnO_2_ nano-particles on nitrogen doped graphene	0.1 M KOH	0.91	0.8	3.9	[69]
10	MnO_2_ nano-particles in three-dimensional graphene–CNT hybrids	0.1 M KOH	0.89	0.72	3.92	This work

## Supplementary Materials

The following are available online at https://www.mdpi.com/2079-4991/10/9/1895/s1, Figure S1: XRD of Mn-H_3_BTC MOF and Mn-H_3_BTC MOF anchored on graphene oxide and carbon fiber; Figure S2: Representative in-lens (top) and its corresponding secondary electron SEM micrograph (bottom) of Mn-H_3_BTC MOF decorated carbon fibers.

## References

[1] Kusano S., Matsumura D., Ishii K., Tanaka H., Mizuki J. (2019). Electrochemical Adsorption on Pt Nanoparticles in Alkaline Solution Observed Using in Situ High Energy Resolution X-ray Absorption Spectroscopy. Nanomaterials.

[2] Morozan A., Jousselme B., Palacin S. (2011). Low-platinum and platinum-free catalysts for the oxygen reduction reaction at fuel cell cathodes. Energy Environ. Sci..

[3] Liu Y., Liu Z., Liu H., Liao M. (2019). Novel Porous Nitrogen Doped Graphene/Carbon Black Composites as Efficient Oxygen Reduction Reaction Electrocatalyst for Power Generation in Microbial Fuel Cell. Nanomaterials.

[4] Kim S., Park H., Li O.L. (2020). Cobalt Nanoparticles on Plasma-Controlled Nitrogen-Doped Carbon as High-Performance ORR Electrocatalyst for Primary Zn-Air Battery. Nanomaterials.

[5] Kim Y., Jeffery A.A., Min J., Jung N. (2019). Modulating Catalytic Activity and Durability of PtFe Alloy Catalysts for Oxygen Reduction Reaction through Controlled Carbon Shell Formation. Nanomaterials.

[6] Liu M., Yu F., Ma C., Xue X., Fu H., Yuan H., Yang S., Wang G., Guo X., Zhang L. (2019). Effective Oxygen Reduction Reaction Performance of FeCo Alloys in Situ Anchored on Nitrogen-Doped Carbon by the Microwave-Assistant Carbon Bath Method and Subsequent Plasma Etching. Nanomaterials.

[7] Verma A., Jha A.K., Basu S. (2005). Manganese dioxide as a cathode catalyst for a direct alcohol or sodium borohydride fuel cell with a flowing alkaline electrolyte. J. Power Sources.

[8] Roche I., Chaînet E., Chatenet M., Vondrák J. (2007). Carbon-Supported Manganese Oxide Nanoparticles as Electrocatalysts for the Oxygen Reduction Reaction (ORR) in Alkaline Medium:  Physical Characterizations and ORR Mechanism. J. Phys. Chem. C.

[9] Osgood H., Devaguptapu S.V., Xu H., Cho J.P., Wu G. (2016). Transition metal (Fe, Co, Ni and Mn) oxides for oxygen reduction and evolution bifunctional catalysts in alkaline media. Nano. Today.

[10] Saito Y., Meguro M., Ashizawa M., Waki K., Yuksel R., Unalan H.E., Matsumoto H. (2017). Manganese dioxide nanowires on carbon nanofiber frameworks for efficient electrochemical device electrodes. RSC Adv..

[11] https://en.wikipedia.org/wiki/Abundance_of_elements_in_Earth’s_crust.

[12] Chen Z., Jiao Z., Pan D., Li Z., Wu M., Shek C.-H., Wu C.M.L., Lai J.K.L. (2012). Recent Advances in Manganese Oxide Nanocrystals: Fabrication, Characterization, and Microstructure. Chem. Rev..

[13] Julien C.M., Mauger A. (2017). Nanostructured MnO_2_ as Electrode Materials for Energy Storage. Nanomaterials.

[14] Sridhar V., Park H. (2019). Manganese nitride stabilized on reduced graphene oxide substrate for high performance sodium ion batteries, super-capacitors and EMI shielding. J. Alloys Compd..

[15] Tang Y., Chen T., Guo W., Chen S., Li Y., Song J., Chang L., Mu S., Zhao Y., Gao F. (2017). Reduced graphene oxide supported MnS nanotubes hybrid as a novel non-precious metal electrocatalyst for oxygen reduction reaction with high performance. J. Power Sources.

[16] Zhan Y., Lu M., Yang S., Xu C., Liu Z., Lee J.Y. (2016). Activity of Transition-Metal (Manganese, Iron, Cobalt, and Nickel) Phosphates for Oxygen Electrocatalysis in Alkaline Solution. ChemCatChem.

[17] Gangaraju D., Sridhar V., Lee I., Park H. (2017). Graphene–carbon nanotube–Mn_3_O_4_ mesoporous nano-alloys as high capacity anodes for lithium-ion batteries. J. Alloys Compd..

[18] Zhang Q., Liu Y.F., Duan Y.D., Fu N.Q., Liu Q.P., Fang Y.Y., Sun Q.W., Lin Y. (2014). Mn_3_O_4_/Graphene Composite as Counter Electrode in Dye-Sensitized Solar Cells. RSC Adv..

[19] Dessie Y., Tadesse S., Eswaramoorthy R., Abebe B. (2019). Recent developments in manganese oxide based nanomaterials with oxygen reduction reaction functionalities for energy conversion and storage applications: A review. J. Sci. Adv. Mater. Devices.

[20] Liu X., Chen C., Zhao Y., Jia B. (2013). A Review on the Synthesis of Manganese Oxide Nanomaterials and Their Applications on Lithium-Ion Batteries. J. Nanomater..

[21] Li L., Nan C., Lu J., Peng Q., Li Y. (2012). α-MnO_2_ nanotubes: High surface area and enhanced lithium battery properties. Chem. Commun..

[22] Ma R., Bando Y., Zhang L., Sasaki T. (2004). Layered MnO_2_ Nanobelts: Hydrothermal Synthesis and Electrochemical Measurements. Adv. Mater..

[23] Xu K., Li S., Yang J., Hu J. (2018). Hierarchical hollow MnO_2_ nanofibers with enhanced supercapacitor performance. J. Colloid Interface Sci..

[24] Ranjith K.S., Raju G.S.R., Chodankar N.R., Ghoreishian S.M., Kwak C.H., Huh Y.S., Han Y.-K. (2020). Electroactive Ultra-Thin rGO-Enriched FeMoO_4_ Nanotubes and MnO_2_ Nanorods as Electrodes for High-Performance All-Solid-State Asymmetric Supercapacitors. Nanomaterials.

[25] Elmacı G., Ertürk A.S., Sevim M., Metin Ö. (2019). MnO_2_ nanowires anchored on mesoporous graphitic carbon nitride (MnO_2_@mpg-C_3_N_4_) as a highly efficient electrocatalyst for the oxygen evolution reaction. Int. J. Hydrogen Energy.

[26] Wan X., Yang S., Cai Z., He Q., Ye Y., Xia Y., Li G., Liu J. (2019). Facile Synthesis of MnO_2_ Nanoflowers/N-Doped Reduced Graphene Oxide Composite and Its Application for Simultaneous Determination of Dopamine and Uric Acid. Nanomaterials.

[27] Li B., Rong G., Xie Y., Huang L., Feng C. (2006). Low-Temperature Synthesis of A-MnO_2_ Hollow Urchins and Their Application in Rechargeable Li+ Batteries. Inorg. Chem..

[28] Kim J., Kim J.S., Baik H., Kang K., Lee K. (2016). Porous β-MnO_2_ nanoplates derived from MnCO_3_ nanoplates as highly efficient electrocatalysts toward oxygen evolution reaction. RSC Adv..

[29] Wang N., Cao X., Lin G., Shihe Y. (2007). λ-MnO_2_ nanodisks and their magnetic properties. Nanotechnology.

[30] Reddy R.N., Reddy R.G. (2003). Sol–gel MnO_2_ as an electrode material for electrochemical capacitors. J. Power Sources.

[31] Abulizi A., Yang G.H., Okitsu K., Zhu J.-J. (2014). Synthesis of MnO_2_ nanoparticles from sonochemical reduction of MnO_4_^−^ in water under different pH conditions. Ultrason Sonochem..

[32] Chen S., Zhu J., Han Q., Zheng Z., Yang Y., Wang X. (2009). Shape-Controlled Synthesis of One-Dimensional MnO_2_ via a Facile Quick-Precipitation Procedure and Its Electrochemical Properties. Cryst. Growth Des..

[33] Walanda D.K., Lawrance G.A., Donne S.W. (2005). Hydrothermal MnO_2_: Synthesis, structure, morphology and discharge performance. J. Power Sources.

[34] Shin J., Seo J.K., Yaylian R., Huang A., Meng Y.S. (2019). A review on mechanistic understanding of MnO_2_ in aqueous electrolyte for electrical energy storage systems. Int. Mater. Rev..

[35] Cheng F., Su Y., Liang J., Tao Z., Chen J. (2010). MnO_2_-Based Nanostructures as Catalysts for Electrochemical Oxygen Reduction in Alkaline Media. Chem. Mater..

[36] Manna B., Ghosh U.C. (2005). Pilot-Scale Performance of Iron and Arsenic Removal from Contaminated Groundwater. Water Qual. Res. J..

[37] Zhang J., Deng P.-H., Feng Y.-L., Kuang Y.-F., Yang J.-J. (2004). Electrochemical Determination of Ascorbic Acid at β-MnO_2_ Modified Carbon Black Microelectrodes. Microchim. Acta.

[38] Chen S., Zhu J., Wu X., Han Q., Wang X. (2010). Graphene Oxide−MnO_2_ Nanocomposites for Supercapacitors. ACS Nano.

[39] Wang H., Peng C., Peng F., Yu H., Yang J. (2011). Facile synthesis of MnO_2_/CNT nanocomposite and its electrochemical performance for supercapacitors. Mater. Sci. Eng. B..

[40] El-Deen A.G., Barakat N.A.M., Kim H.Y. (2014). Graphene wrapped MnO_2_-nanostructures as effective and stable electrode materials for capacitive deionization desalination technology. Desalination.

[41] Zhang Q., Wu X., Zhang Q., Yang F., Dong H., Sui J., Dong L. (2019). One-step hydrothermal synthesis of MnO2/graphene composite for electrochemical energy storage. J. Electroanal. Chem..

[42] Basirun W., Sookhakian M., Baradaran S., Endut Z., Mahmoudian M.R., Ebadi M., Yousefi R., Ghadimi H., Ahmed S. (2015). Graphene oxide electrocatalyst on MnO_2_ air cathode as an efficient electron pump for enhanced oxygen reduction in alkaline solution. Sci. Rep..

[43] Llabrés i Xamena F., Corma A., Garcia H. (2007). Applications for Metal–Organic Frameworks (MOFs) as Quantum Dot Semiconductors. J. Phys. Chem. C.

[44] Chen L.Y., Bai J.F., Wang C.Z., Pan Y., Scheer M., You X.Z. (2008). One-step solid-state thermolysis of a metal–organic framework: A simple and facile route to large-scale of multiwalled carbon nanotubes. Chem. Commun..

[45] Sridhar V., Park H. (2019). Zeolitic imidazolate frameworks as novel precursors for microwave synthesis of carbon nanotubes. J. Alloys Compd..

[46] Arbulu R.C., Jiang Y.-B., Peterson E.J., Qin Y. (2018). Metal–Organic Framework (MOF) Nanorods, Nanotubes, and Nanowires. Angew. Chem. Int. Ed..

[47] Volosskiy B., Niwa K., Chen Y., Zhao Z., Weiss N.O., Zhong X., Ding M., Lee C., Huang Y., Duan X. (2015). Metal-organic framework templated synthesis of ultrathin, well-aligned metallic nanowires. ACS Nano.

[48] Sridhar V., Park H. (2020). Microwave induced transformation of metal organic frameworks into defect rich carbon nanofibers. New J. Chem..

[49] Mofarah S.S., Adabifiroozjaei E., Pardehkhorram R., Assadi M.H.N., Hinterstein M., Yao Y., Liu X., Ghasemian M.B., Kalantar-Zadeh K., Mehmood R. (2019). Coordination Polymer to Atomically Thin, Holey, Metal-Oxide Nanosheets for Tuning Band Alignment. Adv. Mater..

[50] Marcano D.C., Kosynkin D.V., Berlin J.M., Sinitskii A., Sun Z., Slesarev A., Alemany L.B., Lu W., Tour J.M. (2010). Improved Synthesis of Graphene Oxide. ACS Nano.

[51] Tseng L.-T., Lu Y., Fan H.M., Wang Y., Luo X., Liu T., Munroe P., Li S., Yi J. (2015). Magnetic properties in α-MnO_2_ doped with alkaline elements. Sci. Rep..

[52] Elmas Kimyonok A.B., Ulutürk M. (2016). Determination of the Thermal Decomposition Products of Terephthalic Acid by Using Curie-Point Pyrolyzer. J. Energetic Mater..

[53] Montoya Sánchez N., de Klerk A. (2015). Oxidative Ring-Opening of Aromatics: Decomposition of Biphenyl Carboxylic Acids and Zinc Biphenyl Carboxylates. Energy Fuels.

[54] Desimoni E., Brunetti B. (2015). X-ray Photoelectron Spectroscopic Characterization of Chemically Modified Electrodes Used as Chemical Sensors and Biosensors: A Review. Chemosensors.

[55] Jin Y., Zheng Y., Podkolzin S.G., Lee W. (2020). Band gap of reduced graphene oxide tuned by controlling functional groups. J. Mater. Chem. C.

[56] Ganguly A., Sharma S., Papakonstantinou P., Hamilton J. (2011). Probing the Thermal Deoxygenation of Graphene Oxide Using High-Resolution in Situ X-ray-Based Spectroscopies. J. Phys. Chem. C.

[57] Valencia S., Calderón M.J., López-Mir L., Konstantinovic Z., Schierle E., Weschke E., Brey L., Martínez B., Balcells L.I. (2018). Enhancement of spin-orbit coupling at manganite surfaces. Phys. Rev. B.

[58] Şenocak A., Khataee A., Demirbas E., Doustkhah E. (2020). Ultrasensitive detection of rutin antioxidant through a magnetic micro-mesoporous graphitized carbon wrapped Co nanoarchitecture. Sens. Actuators B Chem..

[59] Bernardini S., Bellatreccia F., Casanova Municchia A., Della Ventura G., Sodo A. (2019). Raman spectra of natural manganese oxides. J. Raman Spectrosc..

[60] Rabe M., Toparli C., Chen Y.-H., Kasian O., Mayrhofer K.J.J., Erbe A. (2019). Alkaline Manganese Electrochemistry Studied by in Situ and Operando Spectroscopic Methods-Metal Dissolution, Oxide Formation and Oxygen Evolution. Phys. Chem. Chem. Phys..

[61] Begum H., Ahmed M.S., Jeon S. (2019). δ-MnO_2_ nanoflowers on sulfonated graphene sheets for stable oxygen reduction and hydrogen evolution reaction. Electrochim. Acta.

[62] Li Y., Cao S., Fan L., Han J., Wang M., Guo R. (2018). Hybrid shells of MnO_2_ nanosheets encapsulated by N-doped carbon towards nonprecious oxygen reduction reaction catalysts. J. Colloid Interface Sci..

[63] Guo D., Dou S., Li X., Xu J., Wang S., Lai L., Liu H.K., Ma J., Dou S.X. (2016). Hierarchical MnO_2_/rGO hybrid nanosheets as an efficient electrocatalyst for the oxygen reduction reaction. Int. J. Hydrogen Energy.

[64] Hang Y., Zhang C., Luo X., Xie Y., Xin S., Li Y., Zhang D., Goodenough J.B. (2018). α-MnO_2_ nanorods supported on porous graphitic carbon nitride as efficient electrocatalysts for lithium-air batteries. J. Power Sources.

[65] Chen R., Yan J., Liu Y., Li J. (2015). Three-dimensional nitrogen-doped graphene/MnO nanoparticle hybrids as a high-performance catalyst for oxygen reduction reaction. J. Phys. Chem. C.

[66] Yu Q., Xu J., Wu C., Zhang J., Guan L. (2016). MnO_2_ nanofilms on nitrogen-doped hollow graphene spheres as a high-performance electrocatalyst for oxygen reduction reaction. ACS Appl. Mater. Interfaces.

[67] Ma T., Li C., Chen X., Cheng F., Chen J. (2017). Spinel cobalt–manganese oxide supported on non-oxidized carbon nanotubes as a highly efficient oxygen reduction/evolution electrocatalyst. Inorg. Chem. Front..

[68] Marsudi M.A., Ma Y., Prakoso B., Hutani J.J., Wibowo A., Zong Y., Liu Z., Sumboja A. (2020). Manganese Oxide Nanorods Decorated Table Sugar Derived Carbon as Efficient Bifunctional Catalyst in Rechargeable Zn-Air Batteries. Catalysts.

[69] Mathumba P., Fernandes D.M., Matos R., Iwuoha E.I., Freire C. (2020). Metal Oxide (Co_3_O_4_ and Mn_3_O_4_) Impregnation into S, N-doped Graphene for Oxygen Reduction Reaction (ORR). Materials.

[70] Sridhar V., Jung K.H., Park H. (2020). Vitamin Derived Nitrogen Doped Carbon Nanotubes for Efficient Oxygen Reduction Reaction and Arsenic Removal from Contaminated Water. Materials.

