# Supplementary material for: Metal Organic Framework Derived MnO2-Carbon Nanotubes for Efficient Oxygen Reduction Reaction and Arsenic Removal from Contaminated Water
Source: Nanomaterials (Basel). 2020 Sep 22;10(9):1895. doi: 10.3390/nano10091895 (PMC7558426; doi:10.3390/nano10091895)
Supplement: Supplementary file 1 [file nanomaterials-10-01895-s001.pdf]

Supplementary file for

"Metal Organic Framework derived MnO_2- Carbon Nanotubes for Efficient Oxygen Reduction Reaction and Arsenic Removal from Contaminated Water"

Vadahanambi Sridhar[1], Inwon Lee[2], Kwang Hyo Jung [2] and Hyun Park [1,2*]

[1]Global Core Research Centre for Ships and Offshore Plants (GCRC-SOP), Pusan National University;
[2]Department of Naval Architecture and Ocean Engineering, Pusan National University, Busan 46241, Republic of Korea

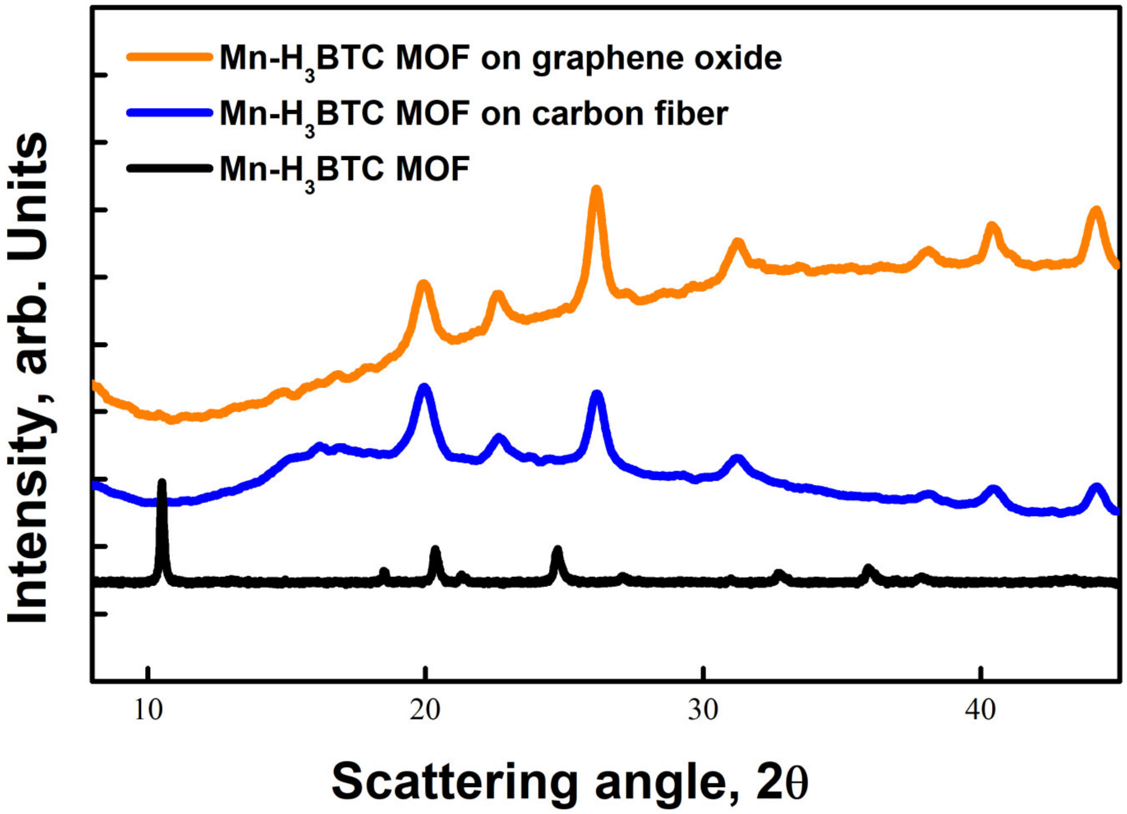

Figure S1 XRD of Mn-H_3BTC MOF and Mn-H_3BTC MOF anchored on graphene oxide and carbon fiber.

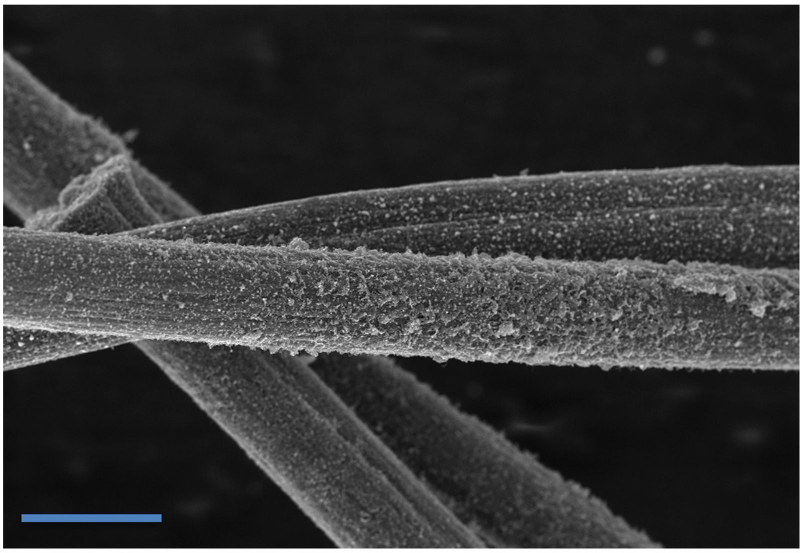

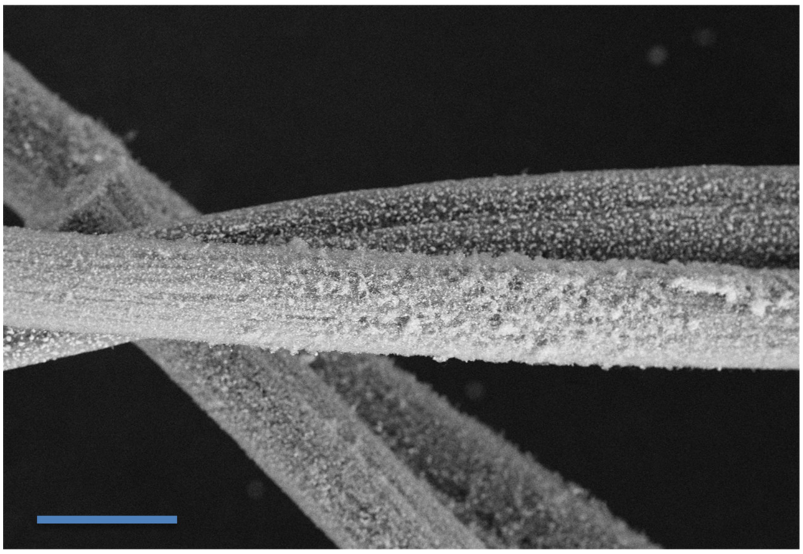

Figure S2 Representative in-lens (top) and its corresponding secondary electron SEM micrograph (bottom) of Mn-H_3BTC MOF decorated carbon fibers. Scale bar is 5 µm in both images.
